# The Effect of Platelet-Rich Fibrin in Preventing Inferior Alveolar Nerve Paresthesia After Mandibular Third Molar Surgery: A Randomized Clinical Trial

**DOI:** 10.1055/s-0046-1816077

**Published:** 2026-03-14

**Authors:** Amirhossein Moaddabi, Gianrico Spagnuolo, Aida Karimzadegan, Shahab Papi, Abolfazl Hosseinnataj, Avideh Maboudi, Ali Semnani, Parisa Soltani, Carlo Rengo

**Affiliations:** 1 Department of Oral and Maxillofacial Surgery, Dental Research Center, Mazandaran University of Medical Sciences, Sari, Iran; 2 Department of Neurosciences, Reproductive and Odontostomatological Sciences, University of Naples “Federico II,” Naples, Italy; 3 Global Research Cell, D. Y. Patil Dental College and Hospital, D. Y. Patil Vidyapeeth, Pimpri, Pune, India; 4 Student's Research Committee, School of Dentistry, Mazandaran University of Medical, Sari, Iran; 5 Department of Geriatric Health, School of Health, Mazandaran University of Medical Sciences, Sari, Iran; 6 Department of Biostatistics, Faculty of Health, Mazandaran University of Medical Sciences, Sari, Iran; 7 Department of Periodontology, Dental Research Center, Mazandaran University of Medical Sciences, Sari, Iran

**Keywords:** third molar, platelet-rich fibrin, paresthesia, inferior alveolar nerve

## Abstract

**Objectives:**

Platelet-rich fibrin (PRF), an autologous biomaterial, has been increasingly utilized to mitigate postoperative pain, edema, and alveolar osteitis, while promoting both soft and hard tissue regeneration during the surgical extraction of third molars. The present study aimed to compare the incidence of postoperative paresthesia in patients undergoing impacted third molar surgery with PRF application versus those treated without PRF.

**Materials and Methods:**

A double-blinded randomized clinical trial was conducted involving patients scheduled for surgical extraction of impacted third molars. Cone-beam computed tomography was utilized preoperatively to confirm the anatomical relationship between the inferior alveolar nerve canal and the tooth roots. Patients were randomly allocated into two groups: the intervention group received PRF placement within the extraction socket, whereas the control group received no adjunctive material. The incidence of postoperative paresthesia was evaluated on the seventh day following surgery using the light-touch sensory test.

**Statistical Analysis:**

Normality was assessed via the Kolmogorov-Smirnov test, and group comparisons were conducted using the Mann–Whitney *U* -test, Pearson's chi-square test, or Fisher's exact test ( *α* = 0.05).

**Results:**

A total of 100 participants were enrolled in the study, comprising 67 females and 33 males, with a mean age of 23.78 ± 3.49 years. There were no statistically significant differences in age or sex distribution between the PRF and control groups ( *p* = 0.464 and *p* = 0.832, respectively). Postoperative paresthesia was observed in 8% and 2% of patients in the control and PRF groups, respectively; however, this difference did not reach statistical significance ( *p* = 0.117).

**Conclusion:**

The findings of this study indicate that PRF did not significantly reduce the incidence of paresthesia following mandibular third molar extraction. Nonetheless, the results underscore the importance of further research to elucidate PRF's potential role in neurosensory recovery and postoperative outcomes.

## Introduction

An impacted tooth is a tooth that fails to emerge properly due to factors like improper positioning, lack of space, or physical obstructions. 1 The third molar, also known as the wisdom tooth, is the tooth most often affected by impaction. Reports show that 73% of young Europeans have an impacted tooth. The mandibular third molar is the most commonly impacted tooth in the mouth, making up 98% of all impaction cases. 2 Keeping an impacted wisdom tooth can cause complications such as pericoronitis (the main reason for extraction), decay in the second or third molar, odontogenic cysts, tumors, periodontitis, chronic orofacial pain, and root resorption. Pericoronitis and decay make up 15% of extraction cases. 3

The surgical extraction of impacted wisdom teeth is one of the most prevalent dental procedures. Due to the proximity of the tooth roots to the mandibular canal and the inferior alveolar nerve (IAN), there exists a risk of nerve injury, rendering it one of the most challenging dental procedures. 4 5 Several standard precautions are used to reduce the risk of paresthesia and nerve injury during this procedure. These include making minimal incisions, avoiding excessive handling of the tissues surrounding the nerve, using cone-beam computed tomography (CBCT) for accurate identification of the tooth and nerve, and administering anti-inflammatory or neuroprotective drugs before and after surgery. The surgeon's skill and experience are essential for nerve protection. These procedures cannot eradicate paresthesia; therefore, other steps must be implemented. 6

Research indicates that lingual nerve injury occurs in 6.5 to 22% of cases, whereas IAN injury occurs in 0.4 to 20.3% of instances. 7 8 9 These injuries generally present as paresthesia (abnormal sensations such as tingling, prickling, or reduced sensation) or anesthesia (total absence of sensation), both categorized as dysesthesia. 10 11 Such issues can cause serious problems, including accidental food spillage, lip or tongue biting, burns from hot drinks, unusual chewing, speech issues, inability to hold saliva, and pain. 12 Paresthesia symptoms typically manifest quickly after the discontinuation of local anesthetic, but in rare cases, they may arise days to months postsurgery. 13 Advanced age and horizontally positioned teeth are two factors that can increase the likelihood of nerve injury. 14 A significant number of individuals are reluctant to have wisdom tooth extraction due to concerns regarding potential neurological complications, which may result in further issues. 15

Platelet-rich fibrin (PRF), a second-generation autologous platelet concentrate, functions as a biodegradable scaffold composed of a fibrin matrix rich in leukocytes, platelets, cytokines, and stem cells. 16 17 PRF is commonly used in dental procedures like implants, periodontal tissue engineering, socket preservation, maxillary sinus augmentation, and wisdom tooth extraction. It yields positive outcomes such as less pain, swelling, alveolar osteitis, and improved healing of soft and hard tissues. 18 In neural regeneration, PRF exhibits notable promise for repairing nerve damage, thanks to its growth factors and capacity to decrease local inflammation. 19 Research on non-human subjects has suggested that PRF can enhance the regeneration of axons and myelin sheaths, thereby improving neuronal function in animal models like rabbits. 20 21 In human clinical studies, the use of PRF in procedures such as IAN lateralization has shown a decrease in the time required for nerve regeneration and an improvement in neurological symptoms. 22 In genioplasty procedures, applying leukocyte-rich PRF accelerated mental nerve sensory recovery within 4 months postsurgery. 23 This advantage comes from PRF's capacity to foster an environment that decreases inflammation and promotes tissue healing.

There is not enough data to confirm whether PRF effectively reduces paresthesia and anesthesia after mandibular third molar surgery involving the IAN. This underscores the necessity for additional research. Therefore, the present study investigates the effect of PRF on reducing the incidence of paresthesia following lower wisdom tooth extraction surgery.

## Materials and Methods

This study was done with approval from the Ethics Committee of Mazandaran University of Medical Sciences, under code IR.MAZUMS.REC.1403.322, and registered in the Iranian Clinical Trials Registry (IRCT) with identification IRCT20241212064029N1. Informed written consent had been obtained from all participants before their inclusion in the study, following the principles of Helsinki.

### Trial Design

A randomized, partially double-blinded clinical trial was designed with one experimental and one control group. The experimental group received PRF in the mandibular third molar extraction site, while the control group did not. The results were reported in accordance with the guidelines of the Consolidated Standards of Reporting Trials.

### Participants, Eligibility Criteria, and Settings

The current study involved patients necessitating surgical extraction of mandibular third molars, with eligibility determined by specific criteria. Participants presented with impacted or partially impacted third molars, possessing at least one root in direct contact with the IAN canal, as verified by CBCT, and were classified as ASA I or II. Exclusions included individuals aged under 18 or over 45 years; those at risk of iatrogenic nerve injury; patients with acute pericoronitis or severe preoperative pain; individuals using antidepressants; smokers exceeding 10 cigarettes daily; those on medications influencing nerve, muscle, or bone repair; patients with horizontally impacted molars with crowns in proximity to the nerve; users of osteoporosis medications such as alendronate; and individuals who underwent radiotherapy or chemotherapy within the preceding year. All 100 enrolled patients completed the study. Patients were consecutively sampled and randomly allocated to intervention or control groups using four-unit permutation blocks. 24

### Intervention

One hundred patients were randomly assigned to either the PRF group ( *n* = 50) or the control group ( *n* = 50). In the PRF group, after surgical extraction of the mandibular third molar by a single oral and maxillofacial surgeon (A.M.), PRF was placed in the socket using forceps. In contrast, no material was placed in the control socket. All patients used 2% chlorhexidine mouthwash (Donyaye Behdasht, Tehran) preoperatively. Anesthesia was achieved via inferior alveolar and long buccal nerve blocks using 2% lidocaine with 1:80,000 epinephrine (Darou Pakhsh, Iran). A triangular flap, extending anteriorly to the mesial of the second molar, was created with a no. 15 blade (Isomed, China; Fig. 1 ). Bone covering the tooth was removed as needed, and the tooth was extracted. Subsequently, after PRF placement in the PRF group, the site was sutured with 3–0 sutures (Supa, Iran). 25 For PRF preparation, 10 mL of the patient's blood was collected in a plain tube by a trained nurse, centrifuged at 3,000 rpm for 10 minutes using an IntraSpin centrifuge (the United States). The PRF layer, located above the red blood cell layer, was carefully extracted and placed in the socket before suturing ( Fig. 2 ). 26

**Fig. 1:**
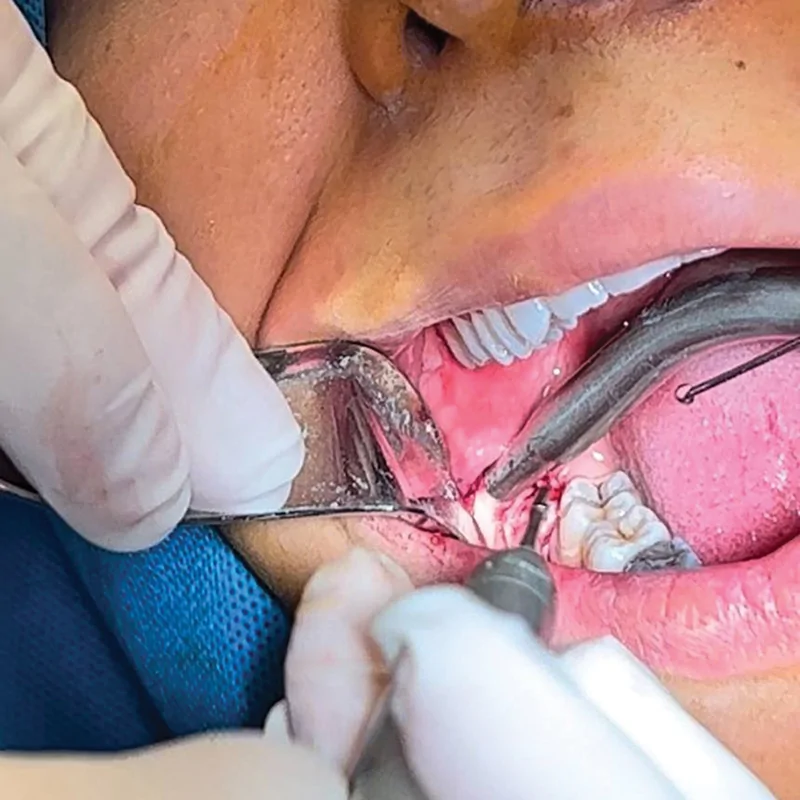
Surgical extraction of the left mandibular third molar.

**Fig. 2:**
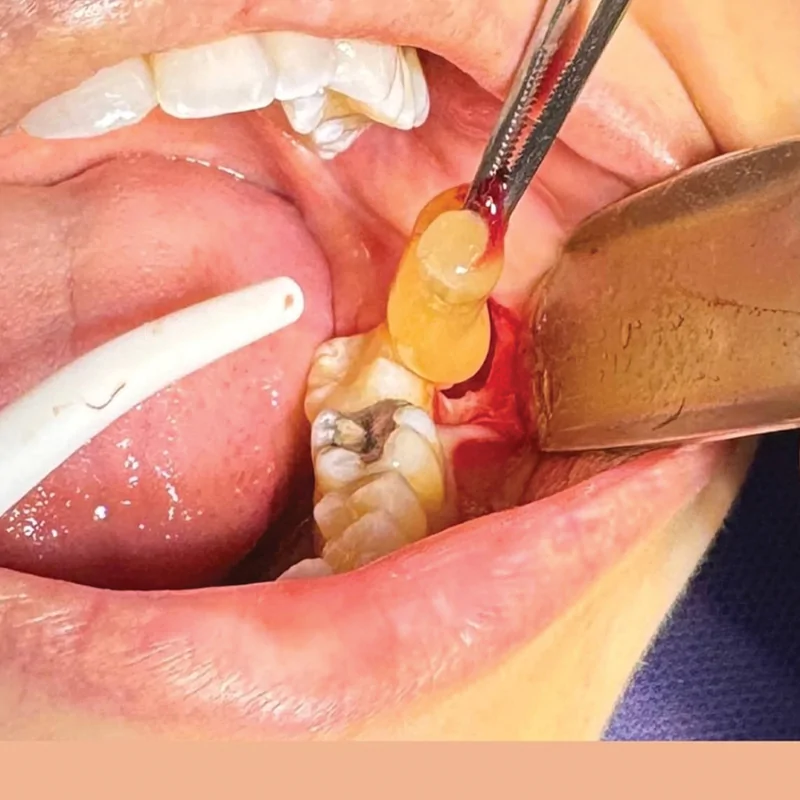
Platelet-rich fibrin placement at the surgical site of the left mandibular third molar.

### Assessment

To determine injury to the IAN 7 days postoperatively and the incidence of postoperative paresthesia following mandibular third molar extraction, light-touch sensation was evaluated using a staircase technique with Semmes–Weinstein monofilaments limited to values below 2.44 mg. 27 28 29 This method was developed to specifically assess light touch sensitivity and to categorize results as either present or absent. All participants were comfortably seated and told to keep their eyes closed throughout the process to reduce visual stimuli. Participants were instructed to raise one hand instantly upon sensing any stimulus at the testing location. 28 Each filament was applied perpendicularly to the skin of the lip and chin with mild pressure until deformation occurred, sustained for approximately 2 seconds, and thereafter removed. The period between applications was standardized to 2 seconds. 27 Each location was presented with a minimum of six genuine stimuli and two blank (placebo) stimuli. Blank stimuli, devoid of filament–tissue interaction, were utilized to identify false-positive responses. Upon a participant reporting sensation during a blank trial, the procedure was stopped, instructions were elucidated, and testing was restarted. The outcome was considered positive if at least one valid stimulus was perceived and negative if no responses were obtained. 27 28 29 Self-reported feelings of the lower lip or mental area were also documented. 29 All assessments have been conducted by a pre-graduate dental student (A.K.).

### Blinding

The trial was conducted with blinding of participants, outcome assessors, and data analysts, while the surgeon was aware of group allocation.

### Statistical Analysis

The present study utilized descriptive data analysis, employing metrics of absolute and relative frequency distribution, mean, and standard deviation. The Kolmogorov-Smirnov test was used to assess the normality assumption. Additionally, demographic and clinical data across groups were evaluated utilizing the Mann–Whitney *U* -test, Pearson's chi-square test, or Fisher's exact test. All statistical analyses were conducted using IBM SPSS version 24. A *p* -value below 0.05 was deemed statistically significant.

## Results

Participant flow is illustrated in the CONSORT flow diagram ( Fig. 3 ). In this study, 67% of the participants were female, and 33% were male. The sex distribution was similar across both groups, and according to the results of Pearson's chi-square test, no statistically significant difference was found between the two groups in terms of sex ( Table 1 ).

**Fig. 3:**
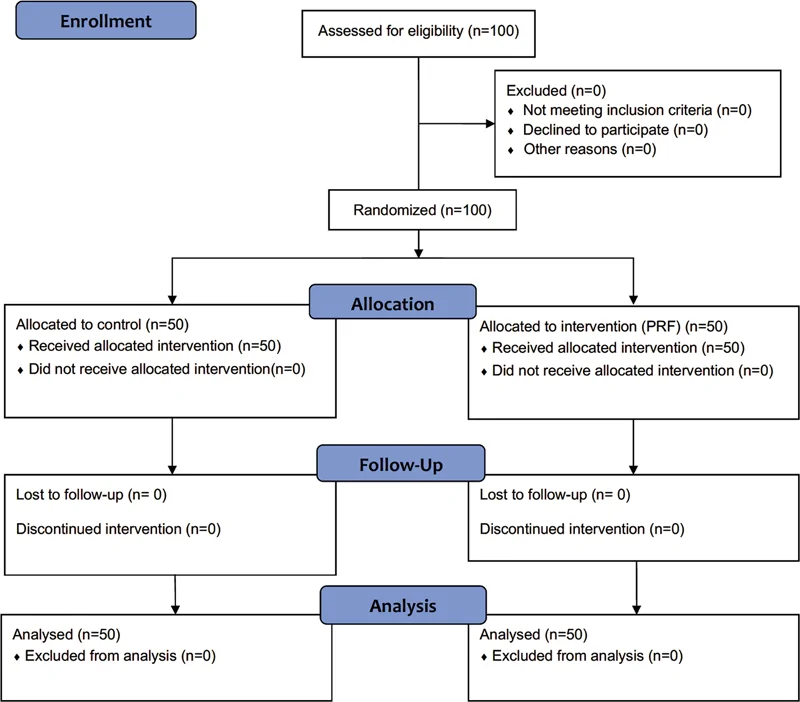
Consort flow diagram. Overview of participant screening, enrollment of 100 eligible patients, random allocation into intervention and control groups, and completion of all study phases.

**Table 1:** Baseline sex distribution of participants in the PRF and control groups

Sex	Control		PRF		*p* -Value
	Count	Percent	Count	Percent	
Female	34	50.7	33	49.3	0.832
Male	16	48.5	17	51.5	

The mean age of all participants was 23.78 ± 3.49 years. In the PRF group, the mean age was 23.30 ± 2.86 years, while in the control group, it was 24.26 ± 3.99 years. Based on the Mann–Whitney *U* -test, no statistically significant difference was observed between the two groups regarding age ( *p* = 0.464).

A comparative analysis of the two groups using Fisher's exact test demonstrated no statistically significant difference in the incidence of paresthesia between the PRF and control groups ( Table 2 ). The results indicated that there were no significant differences between the groups concerning age distribution, sex, occurrence of paresthesia, or recovery time. Although the incidence of paresthesia was observed to be lower in the PRF group relative to the control group (2% vs. 8%), this difference did not reach statistical significance.

**Table 2:** Comparison of postoperative paresthesia incidence between the PRF and control groups at the 7-day follow-up

Paresthesia	Control		PRF		*p* -Value
	Number	Percent	Number	Percent	
No	46	92	49	98	0.117
Yes	4	8	1	2	

## Discussion

Wound healing is a complex biological process involving the participation of multiple cell types and growth factors. Compared with other body systems, regeneration of the nervous system represents one of the slowest and most intricate processes, which does not consistently result in complete functional recovery. 30 The present clinical trial evaluated the effectiveness of PRF in reducing the incidence of paresthesia caused by IAN injury during mandibular third molar extraction procedures in which the nerve was at risk. In this study, although paresthesia was observed in four patients in the control group and one in the PRF group, the difference between groups did not reach statistical significance.

Fibrin is the activated form of a plasma molecule known as fibrinogen, which constitutes the final substrate of all coagulation reactions. Soluble fibrinogen is converted into insoluble fibrin by thrombin, while polymerized fibrin gel forms the initial cicatricial matrix at the injured site. 31 Platelets play a pivotal role in hemostasis and serve as a natural source of growth factors, including platelet-derived growth factor (PDGF), insulin-like growth factor, platelet-derived angiogenesis factor, and vascular endothelial growth factor (VEGF). In addition to growth factors, platelets release numerous other molecules—such as fibronectin, vitronectin, and sphingosine-1-phosphate—that are crucial for wound healing. 32

The PRF preparation protocol was suggested in 2001 by Choukroun and colleagues in France, enabling the entrapment of platelets and cytokines within a fibrin clot. While platelets and leukocyte-derived cytokines contribute significantly to the biological activity of this biomaterial, the fibrin matrix that supports them is considered the key determinant of PRF's true therapeutic potential. 33 PRF represents a fibrin-based biomaterial with a low thrombin concentration, providing an optimal scaffold for endothelial cell and fibroblast migration. This structure facilitates rapid angiogenesis and more efficient fibrin remodeling within resilient connective tissue. Consequently, PRF has been successfully applied in various soft tissue and mucosal repair procedures. 32 34 PRF is obtained through centrifugation of autologous blood without additives under near-physiological conditions, resulting in a dense fibrin matrix capable of sustained release of growth factors. 35 Despite its simple and cost-effective preparation, rapid handling of blood samples is critical, as delayed centrifugation leads to dispersed fibrin polymerization within the glass tube, yielding only a weak clot. 36

In the present study, the incidence of paresthesia in the PRF group following mandibular third molar surgery was 6% lower compared with the control group. The therapeutic effects of PRF are frequently attributed to its growth factor composition (PDGF, VEGF, transforming growth factor-β), which enhances angiogenesis and Schwann cell proliferation, both essential for nerve repair. An experimental study on rats conducted by Huang et al demonstrated that PRF-treated groups exhibited significantly greater myelin sheath thickness and axonal regeneration compared with controls. 37 Similarly, Ikumi and colleagues investigated autografts with and without PRGF in a 15-mm sciatic nerve defect model in rabbits and found that local PRGF application improved axonal diameter and distal axonal regeneration. 38 In line with these findings, Tabrizi et al performed a randomized double-blind clinical trial showing that PRF accelerated IAN recovery after sagittal split osteotomy. 39 Roth et al further reported that PRF-filled venous conduits served as a favorable alternative to autologous nerve grafts in a 1-cm sciatic nerve injury model. 40 Moreover, Şenses et al reported that PRF application enhanced functional recovery in sciatic nerve transection models. 41 By contrast, Lichtenfels et al observed that while PRF promoted functional recovery in a similar model, it did not yield significant improvements in histomorphometric outcomes, 42 while Bayram et al found no histomorphometric or functional benefits of PRF membrane in a rabbit sciatic nerve crush model. 30 Unlike these studies, the present trial did not employ conduits; rather, PRF was placed directly within the surgical socket close to the IAN, potentially accounting for discrepancies in observed outcomes.

In a broader context, researchers have used various methods to resolve neurosensory disturbances. de Oliveira et al, 43 in a study of 125 patients, reported that low-power laser therapy with an infrared beam emission at 808 nm positively influences the recovery of sensitivity following orthognathic or minor oral surgeries. While in another study, laser therapy and the conventional drug treatment were equally effective and both superior to laser acupuncture for the treatment of paresthesia of the IAN after oral surgeries. 44 In another study, Park et al 45 investigated the pharmacological management of trigeminal nerve injuries in a group of 47 patients. The studied pharmacological agents included steroids, nonsteroidal anti-inflammatory drugs (NSAIDs), topical lidocaine, vitamin B complex, adenosine triphosphate, antiepileptics, antidepressants, and opioids, administered alone or in combination for an average of 20.7 weeks. More than half of the patients reported symptomatic improvement, whereas 27.7% showed no significant change and 19.1% had unknown outcomes. Although drug-specific outcomes were not separated, the overall pattern suggests that regimens including antiepileptics and antidepressants were more effective than NSAIDs or opioids alone. In 2025, Vinci et al 19 conducted a systematic review and meta-analysis evaluating adjunctive methods such as piezo-surgery and PRF for minimizing neurosensory complications following IAN transposition and lateralization. The findings indicated that lateralization combined with piezo-surgery and PRF yields favorable outcomes in reducing nerve injury and enhancing implant success.

The findings of this clinical trial are consistent with emerging evidence supporting the neuroprotective and tissue-regenerative potential of PRF. For example, Torul et al demonstrated in a rat model that PRF and PRGF enhanced early nerve regeneration, with PRGF exhibiting superior effects. 35 Malhotra et al further showed that PRF significantly contributed to bone regeneration distal to the mandibular second molar, accelerating both bone and soft tissue healing and thereby reducing the likelihood of postoperative periodontal complications after third molar surgery. 46 Despite the extensive literature on PRF in soft tissue regeneration, relatively few studies have addressed its role in the recovery of paresthesia and anesthesia following surgery. Nevertheless, consistent with the findings of Malhotra et al, the present study provides additional evidence supporting the favorable postoperative outcomes of PRF application in mandibular third molar surgery.

In the present study, the light-touch test was employed to evaluate the occurrence of paresthesia resulting from injury to the IAN in the chin and lower lip region. Alternative neurosensory assessments encompass two-point discrimination, direction discrimination, pinprick, and thermal discrimination tests, as demonstrated in previous research, including the study conducted by Yari et al. 47 In contrast to the aforementioned study, which primarily examined the recovery of the IAN following long-term injuries lasting over 6 months, the current investigation concentrated on identifying new nerve injuries resulting from surgical procedures. Considering the established reproducibility and reliability of the light-touch test in previous studies, along with its documented use in evaluating IAN damage, this method was chosen as the primary tool for neurosensory assessment. 27 28 29

Clinical studies indicate that PRF provides an ideal matrix for stable wound healing without excessive inflammation. PRF, in its platelet gel form, can be used in conjunction with bone grafts, offering multiple advantages such as enhanced wound healing, bone growth and maturation, hemostasis, and improved graft integration. 48

In the current study, a potentially meaningful reduction in paresthesia was observed between the control and PRF groups (8 vs. 2%), prompting the recommendation for future randomized controlled trials with larger sample sizes to enhance statistical power and precision of effect estimates. Additionally, longitudinal assessment of sensory recovery at 1- and 3-month follow-up intervals, as well as extension of the intervention to more neurosensory-risk-prone procedures such as mandibular orthognathic surgery, is strongly proposed to further elucidate the therapeutic potential of PRF in preventing and resolving iatrogenic trigeminal nerve injuries.

## Conclusion

Although PRF did not significantly reduce IAN paresthesia after third-molar surgery, its preventing effect (2% vs. 8% in controls) is clinically promising and warrants larger randomized controlled trials with extended follow-up to confirm this neuroprotective effect.
